# Weight‐bearing pain and implant migration, progressive radiolucencies, radiolucency more than 2 mm and subsidence on radiographs and CT are generally accepted criteria for knee arthroplasty loosening: An international Delphi consensus study

**DOI:** 10.1002/ksa.12419

**Published:** 2024-08-10

**Authors:** George S. Buijs, Arthur J. Kievit, Matthias U. Schafroth, Michael T. Hirschmann, Leendert Blankevoort, International Consensus Panel

**Affiliations:** ^1^ Department of Orthopaedic Surgery and Sport Medicine Amsterdam UMC, Location AMC Amsterdam The Netherlands; ^2^ Amsterdam Movement Sciences Musculoskeletal Health Amsterdam The Netherlands; ^3^ Department of Orthopaedic Surgery and Traumatology Kantonsspital Baselland (Bruderholz, Liestal, Laufen) Bruderholz Switzerland; ^4^ Department of Clinical Research, Research Group Michael T. Hirschmann, Regenerative Medicine & Biomechanics University of Basel Basel Switzerland

**Keywords:** aseptic loosening, clinical signs, consensus, CT, knee arthroplasty, radiographs

## Abstract

**Purpose:**

Establishing the diagnosis of loosening in total or unicondylar knee arthroplasty remains a challenge with different clinical and radiological signs evaluated in study designs with high risk of bias, where few or incomplete criteria are formulated for establishing the diagnosis of implant loosening. This study aimed at evaluating the variability between different clinical and radiological criteria and establish a consensus regarding clinical and radiological criteria for the diagnosis of knee arthroplasty loosening.

**Methods:**

Highly specialized knee surgeons focusing on revision arthroplasty were invited to take part in an international panel for a Delphi consensus study. In the first round, the participants were asked to state their most important clinical and radiological criteria for implant loosening. In a second round, the panel's agreement with the collected criteria was rated on a 5‐point Likert scale (1–5). High variability was defined by receiving at least one score each indicating complete disagreement and complete agreement. Consensus was established when over 70% of participants rated a criterion as ‘fully agree’ (5) or ‘mostly agree’ (4).

**Results:**

High variability was observed in 56% of clinical criteria and 38% of radiological criteria. A consensus was reached on one clinical (weight‐bearing pain [82%]) and four radiological criteria, that is, implant migration, progressive radiolucencies, subsidence and radiolucencies >2 mm on X‐ray or computed tomography (CT) (84%–100%).

**Conclusion:**

Amongst specialized knee revision surgeons, there is high variability in clinical and radiological criteria that are seen as important contributing factors to diagnosis of knee implant loosening. A consensus was reached on weight‐bearing pain as clinical criterion and on implant migration, progressive radiolucencies, subsidence and radiolucencies of more than 2 mm on X‐ray or CT as radiological criteria. The variability rates observed, along with the criteria that reached consensus, offer important insights for the standardization of diagnostic protocols.

**Level of evidence:**

Level V.

**Abbreviations**
AP: anteroposterior
CT: computed tomography
MRI: magnetic resonance imaging
rTKA: revision TKA
SPECT/CT: single‐photon emission computerized tomography and computed tomography
TKA: total knee arthroplasty
UKA: unicondylar knee arthroplasty

## INTRODUCTION

After total knee arthroplasty (TKA) and unicondylar knee arthroplasty (UKA), the chance for revision TKA (rTKA) within 10 years are approximately 13% and 12%, respectively [13]. The absolute number of rTKA will increase with the upward trend of numbers of primary TKA and UKA, despite the improvements of TKA and UKA designs and surgical procedures [11, 12, 13, 16].

As 20%–30% of the revisions are performed due to aseptic loosening, it stands as a significant cause of TKA failure [5, 19]. Aseptic loosening often requires complex revision surgery, heavily impacting patients and healthcare systems worldwide [9, 10]. A correct diagnosis is essential, as unnecessary revision surgery should be avoided for patients incorrectly diagnosed with TKA loosening. Alternatively, undetected TKA loosening due to an inadequate diagnosis may lead to patients being erroneously denied revision surgery.

In the quest to enhance diagnostic accuracy for aseptic loosening, numerous studies have investigated various diagnostic criteria and modalities [1, 2, 3, 18, 20]. However, these studies have frequently yielded conflicting outcomes, contributing to a significant degree of heterogeneity of diagnostic criteria in meta‐analyses [3, 4, 20]. This inconsistency is partly due to the diverse diagnostic techniques employed to establish the diagnosis of aseptic loosening. The diagnosis is based on clinical assessment, clinical laboratory tests and various imaging modalities. Different criteria are used to arrive at the diagnosis [1, 2, 3, 4, 18, 20].

Variations in patient demographics, in the duration of follow‐up and in definitions of which components can be seen as loose intraoperatively, which then is used as reference standard have compounded the complexity of drawing conclusive inferences [3]. As a result, the orthopaedic community still faces challenges in establishing a standardized diagnostic protocol for aseptic loosening, which is essential for guiding treatment decisions and improving patient outcomes [22].

Amidst all these uncertainties and challenges, no study has yet explored the extent of variability and consensus of the clinical and radiological criteria for the diagnosis of aseptic loosening in knee arthroplasty. In the absence of any consensus based on literature data, a Delphi study might help to arrive at a consensus for future use in clinical studies and in daily practice. It was hypothesized that high variability would be observed for several clinical and radiological criteria. No hypothesis was formulated for criteria expected to reach consensus. The aim of this study is to answer the following research questions by using the Delphi Consensus method:

(1) *What is the variability in the perceived trustworthiness of preoperative clinical and radiological criteria used to diagnose aseptic knee arthroplasty loosening?*
(2) *Which preoperative clinical and radiological criteria are considered trustworthy by specialized knee revision experts to establish a consensus for diagnosing aseptic knee arthroplasty loosening?*

## MATERIALS AND METHODS

For the current Delphi consensus study, the methodological criteria and reporting guidelines as recommended by Diamond et al. and Jünger et al. were employed [6, 8]. After the international consensus panel had been assembled, the Delphi study unfolded over two rounds. The first round focused on identifying clinical and radiological criteria that, according to knee revision specialists, are associated with aseptic loosening in knee arthroplasty. The second round involved rating the significance of these identified criteria. If a consensus was not reached within the second round, a third round was available to rerate the importance of criteria after having seen a summary of the results from the second round. This study only considered loosening of the tibial and femoral components.

The lead author (G. S. B.) acted as the coordinator, responsible for crafting the questionnaires based on participant responses and managing all communications. To avoid moderator bias by influencing the study's outcome, the lead author did not participate in the study as a panel member. Co‐authors M. U. S., A. J. K. and M. T. H. did participate as panel members, but did not participate in the processing and analysis of the responses.

### Assembling the international consensus panel

Orthopaedic surgeons specialized in knee arthroplasty revision were identified in three different ways; (1) through screening of author lists of high‐quality articles dealing with the diagnosis of aseptic loosening in knee arthroplasty, (2) through screening of programmes of major orthopaedic congresses to identify keynote speakers presenting on knee arthroplasty and/or loosening related subjects and (3) through the clinical network of the co‐authors. These three different approaches were adopted to ensure a diverse, international, representative group of panel participants and to potentially reduce selection bias.

To date, there are no definitive guidelines or recommendations regarding the ideal sample size for Delphi studies. There is no clear definition of what constitutes a too small or too large group. While some researchers suggest that a group of 10–15 experts may suffice for homogeneous groups, a larger sample is often recommended when a larger variety is expected [21]. For this study, authors required an *a priori* minimum of 25 participants. In anticipation of non‐responders, a total of 69 potential expert panel participants were identified and invited. All experts who consented to a complete participation were sent the Delphi consensus questionnaires.

### Baseline

At first, all participants were asked to state their years of experience as knee revision surgeon and the average number of knee revision surgeries performed per year. Furthermore, sex and country of current practice were registered. Participants were asked if they wanted to be acknowledged for full participation. An acknowledgement as a group author was only granted if a participant successfully completed all consensus rounds.

### First round

To gain a better understanding of the different opinions between the participants, the following two questions were asked:

(1) *To what extent do you agree that there is no clear uniform specification in available literature of preoperative signs associated with knee arthroplasty component loosening?*
(2) *To what extent do you agree that there is a need for a uniform specification of which preoperative signs are indicative for knee arthroplasty component loosening?*

Both questions were Single Select Multiple Choice Questions using a 5‐point Likert scale (1 being *I fully agree*, 2: *I mostly agree*, 3 *neutral*, 4; *I do not agree completely* and 5; *I do not agree at all*).

The first round identified criteria associated with implant loosening of both the tibial and femoral component of a TKA, UKA or rTKA, separately. Therefore, at first, two yes or no questions were posed:

(1) *In your opinion are there any preoperative clinical signs that are associated with loosening of the tibial component (partial/primary and/or secondary)?*
(2) *In your opinion are there any preoperative clinical signs that are associated with loosening of the femoral component (partial/primary and/or secondary)?*

If either of the questions were answered with ‘yes’, participants were asked to specify these elements for UKA, TKA and rTKA separately in two separate open questions. One question regarding the loosening of the tibial component and the second question with regard to loosening of the femoral component.

### Second round and third round

In preparation for the second round, all statements were collected and duplicates were removed. All statements were incorporated in the second‐round survey *as* ‘“statement” is indicative for aseptic loosening of all components of either partial, primary or revision knee arthroplasties. As no distinctive different elements were registered in the first round, all statements were presented as applicable to UKA, TKA and/or rTKA and for both the tibia and femur components. Participants were asked to rate their agreement with the statements according to a 5‐point Likert scale.

Results of the second round were summarized in frequency tables and presented to all participants. High variability regarding a particular statement was defined as the statement having received at least 1 score of complete disagreement and at least 1 score of complete agreement, both must apply. Consensus was defined as >70% of participants rating the statement either as ‘I fully agree’ or ‘I mostly agree’. No third round took place because consensus was already achieved in the second round.

### Data collection and analysis

Baseline demographic data were presented as averages and standard deviations or medians and interquartile ranges according to their distribution. These included years of experience, surgical volume and country of residence. The categorical variables ‘consensus’ were expressed as absolute numbers and percentages. If a participant failed to complete the first round, he or she was excluded from further participation and statistical analysis. The *χ* ^2^ test was used to evaluate potential differences between Dutch responders and non‐Dutch responders for those statements that received consensus. A *p* < 0.05 was considered statistically significant. Data were analyzed using Excel (Microsoft Excel 2018; Microsoft Corp.) and Python (Python 3.8, Python Software Foundation). Questionnaires were distributed using Castor EDC, an online platform for questionnaire dissemination and data collection.

## RESULTS

### Panel characteristics

**Figure 1:**
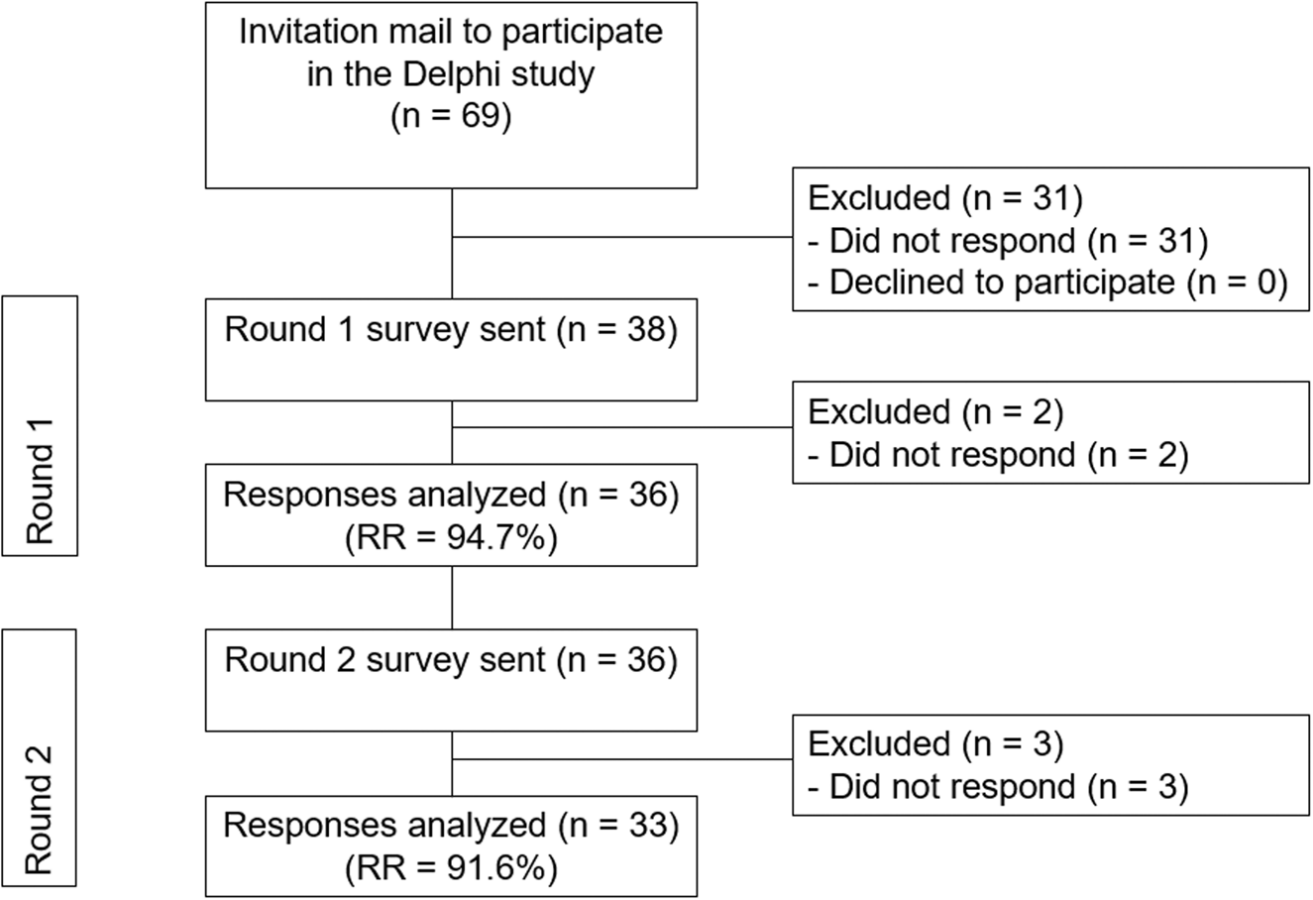
Flowchart of inclusion of panel members. Flowchart of inclusion of panel members and response rates (RRs) per round.

A total of 69 eligible expert panel participants were contacted and 38 (55.1%) agreed to participate and were therefore included in this Delphi consensus study. All but two (*n* = 36; 94.7%) who agreed to participate completed the first round. As four participants failed to complete the second round, both rounds were completed by 33 (84.2%) participants (Figure 1).

Dutch orthopaedic surgeons were the predominant group, making up 24.2% of participants, the majority of whom were male (93.9%) and performed over 20 knee arthroplasty revision surgeries annually. Most participants (81.8%) boasted over 10 years of experience with knee arthroplasty revisions (Table 1).

**Table 1:** Characteristics of panel members.

Characteristics	*n* = 33	Percentages
Sex		
Male	31	93.9
Female	2	6.1
Country/region of practice		
The Netherlands	8	24.2
United States	4	12.1
Germany	4	12.1
United Kingdom	4	12.1
Belgium	3	9.1
Switzerland	2	6.1
France	2	6.1
India	2	6.1
Indonesia	1	3.0
Peru	1	3.0
Türkiye	1	3.0
Spain	1	3.0
Experience as knee arthroplasty revision surgeon (year)		
0–5	2	6.1
5–10	4	12.1
10–15	11	33.3
20–25	9	27.3
>25	7	21.2
Average knee arthroplasty revision surgeries per year		
0–10	1	3.0
10–20	2	6.1
20–30	8	24.2
30–40	4	12.1
40–50	6	18.2
>50	12	36.4

*Note*: Baseline characteristics of participants completing all rounds of this Delphi consensus study (*n* = 33).

### First round

Amongst the 36 responders in the first round, 26 (75.0%) agreed fully or mostly with the statement that there is no clear uniform specification in available literature of preoperative signs indicative of knee arthroplasty component loosening. The majority (*n* = 34; 94.4%) either fully agreed or mostly agreed to the statement that there is a need for a clear uniform specification of preoperative signs indicative of knee arthroplasty component loosening.

All responders confirmed the existence of preoperative clinical signs that are associated with loosening of either the tibial or femoral component of a UKA, primary TKA or rTKA. All responders generated a total of 28 statements regarding the UKA tibial component, 27 statements with regard to the tibial component of a primary TKA, and 28 statements with regard to the tibial component of an rTKA. Regarding the femoral component, 29 statements concerning UKA, primary TKA and rTKA were proposed. No technically relevant differences were found between statements regarding either UKA, primary TKA or rTKA. Discrimination between these knee arthroplasty types was not further considered.

All statements were then summarized and dichotomized between clinical signs and radiological signs. This resulted in 18 clinical statements (Table 2a). Summarizing the mentioned radiological signs resulted in 16 statements (Table 2b).

**Table 2a:** Level of agreement with clinical statements.

Statements	Percentages of agreement (fully and mostly agreed)
Weight‐bearing pain	81.8^a^
Change in limb alignment	63.6^b^
Pain during axial loading, which decreases after a few steps and then increases again	60.6^b^
Startup pain	57.6^b^
Femoral shaft pain (for femur components of revision arthroplasties)	51.5^b^
Tibial shaft pain (for tibia components of revision arthroplasties)	48.5^b^
Pain on varus‐valgus stress	45.5
Transient swelling around the knee after ambulation	36.4^b^
Tenderness on palpation of the distal thigh *(for femoral components)* and at proximal tibia *(for tibial components)*	33.3
Persistent swelling around the knee	30.3^b^
Antalgic gait	30.3^b^
(Progressive) unloaded pain	24.2^b^
Ligament pseudo laxity	21.2
Pain on palpation of the joint line	21.2
Instability of the knee	12.1
Instability of the knee at flexion or extension	9.1
Knee stiffness	6.1
Peri‐patellar swelling	3.0

*Note*: Level of agreement with statements concerning clinical signs.

a Preset threshold for consensus was met.

^b^ Preset threshold for high variability were met.

**Table 2b:** Level of agreement with radiological statements.

Statements	Percentages of agreement (fully and mostly agreed)
Obvious implant migration on X‐ray or CT	100.0^a^
Progressive radiolucencies on AP and lateral X‐ray or CT	84.8^a^
Subsidence on X‐ray or CT	93.9^a^
Radiolucency on AP and lateral X‐ray or CT > 2 mm around implant or bone‐cement interface	90.9^a^
Continuous fluid film around the implant on MRI	51.5
Activity around the implant on PET‐CT > 2 years after implantation	36.4^b^
Cyst formation on X‐ray or CT	48.5^b^
Cortical thickening at the tip of the stem (for revision arthroplasties)	48.5^b^
Radiolucencies on AP and lateral X‐ray or CT < 2 mm around implant or bone‐cement interface	27.3
Deformity on X‐ray or CT	33.3^b^
Activity around the implant on SPECT‐CT > 2 years after implantation	51.5^b^
Activity around the implant on bone scintigraphy >2 years after implantation	36.4^b^
Increased bone density on X‐ray or CT	21.2
Activity around the implant on bone scintigraphy <2 years after implantation	6.1
Activity around the implant on SPECT‐CT < 2 years after implantation	6.1
Activity around the implant on PET‐CT < 2 years after implantation	0.0

*Note*: Level of agreement with statements concerning radiological signs.

Abbreviations: AP; anterior‐posterior; CT, computed tomography, PET‐CT; positron emission tomography‐computed tomography; SPECT‐CT, single‐photon emission computed tomography‐computed tomography.

a Preset threshold for consensus was met.

^b^ Preset threshold for high variability were met.

### Second round

After being presented the summary of round one, the expert panel participants reached consensus on one clinical and four radiological signs (Table 2a, 2b).

As clinical sign, ‘weight‐bearing pain’ was either fully or mostly agreed upon by 81.8% of the panel experts to be associated with loosening of either the tibial or femoral component in UKA, primary TKA or rTKA. Seven participants (21.2%) fully agreed. High variability was observed in 10 out of 18 (55.6%) statements (Table 2a). ‘Weight‐bearing pain’ did not meet the criterion for high variability.

Four radiological signs that surpassed the preset threshold for consensus were (1) obvious implant migration on X‐ray or CT with 100% agreement [23 (69.7%) fully agreed], (2) progressive radiolucency on anterior‐posterior (AP) and lateral X‐ray or CT with 84.8% of agreement [14 (42%) fully agreed], (3) subsidence on X‐ray or CT with 93.9% of agreement [15 (45.5%) fully agreed] and (4) radiolucency on AP and lateral X‐ray or CT > 2 mm around the implant or bone–cement interface with 90.9% of agreement [13 (39.4%) fully agreed]. High variability was observed in six of 16 (37.5%) statements (Table 2b). None of the statements with more than 70% agreement met the criteria for high variability.

### Subanalysis based on country of practice

Experts from 13 countries participated, with 24.2% from The Netherlands and the remainder from various other countries. No significant differences were between Dutch and other participants for the criteria that met the consensus threshold (Table 3).

**Table 3:** Agreement dichotomized based on country of practice.

Statements	Number of participants that mostly and fully agreed (the Netherlands) (*n* = 8)	Number of participants that mostly and fully agreed (other countries) (*n* = 25)	*p* Value
Clinical			
Weight‐bearing pain	7	20	0.540
Radiological			
Obvious implant migration on X‐ray or CT	8	25	1.000
Progressive radiolucencies on AP and lateral X‐ray or CT	5	23	0.729
Subsidence on X‐ray or CT	8	23	0.525
Radiolucencies on AP and lateral X‐ray or CT > 2 mm around implant or bone–cement interface	6	24	0.250

*Note*: Fully and mostly agree scores for statements receiving consensus dichotomized into groups based on country of practice (the Netherlands vs. other countries).

Abbreviations: AP, anterior‐posterior; CT, computed tomography.

## DISCUSSION

The most important finding of the present study was that consensus was achieved on several key preoperative clinical and radiological criteria for diagnosing aseptic loosening in knee arthroplasty. Notably, weight‐bearing pain was identified as the clinical sign meeting the preset consensus threshold. Regarding radiological signs, consensus was reached on four specific criteria: evident implant migration, progressive radiolucency, subsidence and radiolucency exceeding 2 mm around the implant or bone–cement interface. Nevertheless, the study revealed considerable variance in expert views, with high variability observed in 55.6% of the clinical statements and 37.5% of the radiological statements. These results emphasize the diverse importance attributed to different clinical and radiological findings, yet they also underscore a general agreement amongst international experts on certain crucial criteria. Both the observed high variability rates and the criteria that met the consensus threshold should be considered when establishing diagnostic protocols.

Patients with aseptic loosening initially present with post‐TKA knee pain [15, 17]. Usually, the standard approach encompasses taking of patient history (presence of weight‐bearing pain, minimal pain at full range of motion), physical examination and conventional knee radiography. Nevertheless, the sensitivity and specificity of plain radiography often prove insufficient, particularly in instances of early and subtle yet clinically significant loosening [7, 14, 22].

While these observations are corroborated by the experts in this Delphi consensus, what this study primarily reveals is the lack of consensus and high variability amongst experts regarding the importance they place on the outcomes of advanced diagnostic modalities such as hybrid single‐photon emission computerized tomography and CT (SPECT/CT), magnetic resonance imaging and bone scintigraphy. This is somewhat in contrast to results of published articles indicating high sensitivity and specificity for SPECT/CT as a diagnostic tool for knee arthroplasty loosening in specialized centres [1, 2, 3]. The high variability and lack of consensus regarding these advanced modalities in the Delphi consensus study underscores the need for further prospective studies evaluating advanced diagnostic modalities with a greater number of patients, as limitations reported for these modalities leave room for diverse opinions and preferences.

This study has several limitations, and, therefore, its findings should be interpreted considering the following remarks. First, there is no consensus on the most optimal design of a Delphi consensus study. More rounds or an open discussion of results providing statements with nuances and explanations could potentially have increased or broadened the consensus. Nevertheless, this study was conducted based on prespecified design based on generally accepted methodological criteria [6, 8]. Second, despite the first author's efforts to assemble a well‐represented international panel, this was not fully achieved European (particularly Dutch) and American experts are overrepresented within the panel, but a subanalysis revealed no statistically significant differences for statements that received consensus. And finally, only orthopaedic surgeons were involved in this Delphi consensus. Although a definitive diagnosis is made by orthopaedic surgeons, diagnosing loosening often requires a multidisciplinary approach. The involvement of radiologists and nuclear physicians may very well have altered the current results.

## CONCLUSION

In conclusion, high variability exists amongst expert knee revision surgeons regarding the clinical and radiological standards deemed important for identifying the loosening of knee arthroplasty components. Consensus was reached on weight‐bearing pain as clinical criterion and on implant migration, progressive radiolucency, subsidence and radiolucency >2 mm on radiographs or CT as radiological criteria. Both the observed variability rates and the criteria that achieved consensus provide valuable insights to be considered when standardizing diagnostic protocols.

## AUTHOR CONTRIBUTIONS

**George S. Buijs**: Conceptualization; data curation; formal analysis; investigation; methodology; project administration; resources; supervision; validation; visualization; writing—original draught; writing—review and editing; given final approval. **Arthur J. Kievit**, **Matthias U. Schafroth**, **Michael T. Hirschmann** and **Leendert Blankevoort**: Conceptualization; methodology; supervision; writing—review and editing; given final approval.

## Members of the International Consensus Panel

Adolph Joseph Yates Jr, Antonia F. Chen, Arthur J. Kievit, Arun Mullaji, Benjamin V. Bloch, Bert Boonen, Carsten Perka, Claudia Arias, Daniel C. Wascher, Dr. Kailash Patil, Emmanuel Thienpont, Enrique Gómez‐Barrena, Heiko Graichen, Hermes H. Miozzari, James A. Harty, Jan Victor, Jean‐Yves Jenny, Johannes Beckmann, Jonathan Vigdorchik, Lucien Keijser, Matthias U. Schafroth, Michael T. Hirschmann, Nicolaas C. Budhiparama, Peter C. M. Verdonk, Phil Walmsley, Reha N. Tandogan, Roel Hendrickx, Rutger van Geenen, RWTM van Kempen, Rüdiger von Eisenhart‐Rothe, Sam I.S. Oussedik, Sébastien Lustig, Simon van Laarhoven.

## CONFLICT OF INTEREST STATEMENT

Arthur J. Kievit, Matthias U. Schafroth and Leendert Blankevoort are listed as inventors on a patent for a loading device that can be used to quantify and visualize implant displacement. The remaining authors declare no conflict of interest.

## ETHICS STATEMENT

The authors have nothing to report.

## Data Availability

The data that support the findings of this study are available on request from the corresponding author.
